# X-ray examination of a tissue-engineered epithelial sheet during transportation

**DOI:** 10.3389/fbioe.2026.1764810

**Published:** 2026-03-03

**Authors:** Xiaomin Shao, Fuyue Wu, Zheng Lin Tan

**Affiliations:** ReMed Regenerative Medicine Clinical Application Institute, Shanghai, China

**Keywords:** low-dose X-ray exposure, safety of transportation, tissue-engineered epithelial sheet, tissue-engineered grafts, X-ray irradiation

## Abstract

Autologous tissue-engineered epithelial sheets (TEESs) are generally transported by dedicated couriers. The cost of this technology can be reduced by using cold-chain courier services, in which the TEESs are subjected to X-rays during security checks. We exposed TEESs to a widely used X-ray luggage-control system to the maximum dose limited by regional regulations. DNA fragmentation, unique variants by exome sequencing, and proliferative capabilities were not altered after X-ray exposure. Thus, repeated exposure to X-ray radiation of luggage-control systems did not induce changes in TEESs genetically or biologically, which should simplify the transport of grafts.

## 1 Introduction

Tissue-engineered products, particularly tissue-engineered epithelial sheets (TEESs), are increasingly used for various clinical purposes, for example, corneal reconstruction (Nishida et al., 2004), urethral reconstruction (Karapanos et al., 2021), and treatment of vitiligo lesions (Li et al., 2024). While it remained uncertain whether a low dose of X-ray exposure would affect these epithelial sheets, care has been taken to prevent X-ray exposure when transporting these products. Thus, thousands of grafts are carefully transported by dedicated courier services to prevent X-ray exposure. This requirement not only increases logistics costs but also limits the deliverable distance of grafts.

The dose of X-ray emitted from luggage-control systems is strictly controlled by regional regulation. For example, the Chinese Standard has required that an X-ray dose emitted from X-ray luggage-control systems should not exceed 5 μGy (State Administration for Market Regulation and the Standardization Administration of the People’s Republic of China, 2019), while in the United States of America, a screening dose is limited to 25 μrem (0.25 μSv, equivalent to 0.25 μGy when the quality factor is 1) for general use (Legislative Reference Bureau Commonwealth of Pennsylvania, 2023). Usually, X-rays emitted by a luggage-control system are approximately 0.1–0.2 times the maximum dose limited by regional regulation. If the limited doses are considered safe by respective countries, then the X-rays emitted by a luggage-control system that has a lower dose should also be considered safe.

Interestingly, no data have been published about the presumed adverse effect of X-rays emitted from luggage-control systems on the viability and proliferative capacity of tissue-engineered products to support transportation of TEESs by cold-chain courier services, for which X-ray examination is usually required. This study investigated the effect of X-ray radiation on TEESs to explore the possibility of transporting them with a cold-chain courier service.

## 2 Methods

### 2.1 X-ray irradiation of a tissue-engineered epithelial sheet

TEESs were cultured from cells obtained from patients who underwent autologous cultured epithelial grafting based on a serum-free and feeder-free culture system, as described in our previous study (Lian et al., 2024). This study was conducted based on cells remaining as a by-product after preparing autologous cultured epithelial grafts for the treatment of vitiligo. As the samples were precious, given the limited proliferative capability of epithelial-derived cells, which include keratinocytes and melanocytes, this study was conducted using epidermal-derived cells from three independent patients. The identity of patients was anonymous due to data management policy and because it is irrelevant to this study.

Then, the TEESs were cut into six equal portions by area, and three portions from each piece were subjected to X-ray irradiation with the luggage-control system at Pujiang Town Station, Shanghai (Shanghai Gaojing Detection Technology Co., Ltd., GJ-XS6550, Shanghai, People’s Republic of China, 0.534 μGy per inspection), by passing the TEESs through the machine 1, 3, 5, and 10 times to achieve 0.534 μGy, 1.602 μGy, 2.670 μGy, and 5.34 μGy X-ray irradiation. The remaining three portions were used as a control. Three biological replicates were secured in each test. Because the samples in the experimental group are also the samples in the control group, paired analysis can be conducted.

### 2.2 Detection of DNA breakage

X-ray irradiation-induced DNA fragmentation was detected by a terminal deoxynucleotidyl transferase-mediated deoxyuridine triphosphate nick-end labeling (TUNEL) assay (Naselli et al., 2024).

After irradiation, TEESs were transferred to Hank’s balanced salt solution (HBSS) without calcium and magnesium, followed by incubation in a 37 °C, humidified 5.0% CO_2_ incubator for 2 h. The control was prepared by treating TEESs with a similar treatment but without X-ray irradiation. Then, the TEESs were fixed with 10% formalin solution, labeled with the TUNEL assay (Yeasen Biotechnology, 40306ES50, Shanghai, People’s Republic of China), and counterstained with Hoechst 33528. The positive control was prepared by treating a TEES without X-ray irradiation with DNase I prior to staining, while the negative control was prepared by replacing the TdT enzyme with water. Each piece of stained TEES was observed with a fluorescence microscope (Olympus, IX73, Tokyo, Japan), and the fluorescence intensity at excitation/emission at 346/460 nm for the Hoechst 33528 dye and 485/520 nm for the TUNEL assay of each sample was measured with a spectrophotometer (Thermo Fisher Scientific, Varioskan Lux, Singapore). The statistical differences between the X-ray irradiated samples and the control were analyzed using Student’s t-test and paired t-test.

### 2.3 Whole-exome sequencing of TEESs

TEESs with and without 5.340 μGy X-ray irradiation were incubated in a 37 °C, humidified 5.0% CO_2_ incubator for 30 min. Then, the samples were snap-frozen with liquid nitrogen before DNA extraction and whole-exome sequencing. Whole-exome sequencing was conducted by OE Biotech, Shanghai. In brief, genomic DNA was isolated from the TEES and underwent quality control. Only genomic DNA that passed quality control was used in the subsequent procedure. A total of 200 ng of genomic DNA from each sample was used for whole-exome library preparation with Agilent SureSelect Human All Exon V8 library as per the manufacturer’s instructions. In this process, DNA was sheared with Covaris into 150–200 bp fragments, followed by ligation of adapters and amplification with polymerase chain reaction. The amplified libraries were hybridized to the probes, and the qualified next-generation sequencing libraries were subjected to paired-end sequencing with the Illumina sequencing platform (NovaSeq 6000).

Raw sequencing reads from sequencers were obtained in fastq formats and analyzed with a bioinformatics pipeline to detect genomic alterations. The range of raw base was 20.69 G ∼ 24.12 G data. The sequence data were aligned with the human reference genome (GRCh37.p13) using Burrows–Wheeler Aligner (BWA) (Li and Durbin, 2009), version 0.7.17. The format was converted with Samtools (Li et al., 2009), and the redundancy was removed with Picard (Broad Institute, 2025). The result was compared using Qualimap software. GATK version 4.1.9.0 (McKenna et al., 2010) was employed to recalibrate the base quality score and realignment of single-nucleotide polymorphisms and insertions/deletions. To reduce the possibility of false positivity, the standard for data filtering was set to QD ≥ 2.0. A paired comparison was conducted by comparing the results obtained from an X-ray irradiated sample to its counterpart obtained from the same piece of TEES without X-ray irradiation. Single-nucleotide polymorphisms and insertions/deletions found on both X-ray irradiated samples and control samples were sorted.

### 2.4 Cell viability assay

TEESs before and after X-ray radiation were treated with 0.05% trypsin–ethylenediaminetetraacetic acid (Gibco, 252-00056, NY, United States) diluted with D-PBS (−) for 30 min. Then, the reaction was inhibited with fetal bovine serum, rinsed with D-PBS (−), and suspended in a serum-free, feeder-free culture system. The cells were seeded into a 24-well plate and incubated in a 37 °C, humidified 5.0% CO_2_ incubator for 7 days. The medium was replaced every other day. Then, the cell viability was assessed with cell counting kit-8 (Beyotime, C0038, Shanghai, People’s Republic of China) according to the manufacturer’s instructions. The statistical differences between the X-ray irradiated samples and the control were analyzed using Student’s t-test and a paired t-test.

## 3 Results

### 3.1 X-ray irradiation does not induce DNA fragmentation and apoptosis

One of the most lethal effects caused by X-ray irradiation is DNA fragmentation. DNA fragmentation will, in turn, result in unexpected mutations due to errors in its repair process. Therefore, it is important to confirm DNA integrity in cells after X-ray irradiation.

**FIGURE 1:**
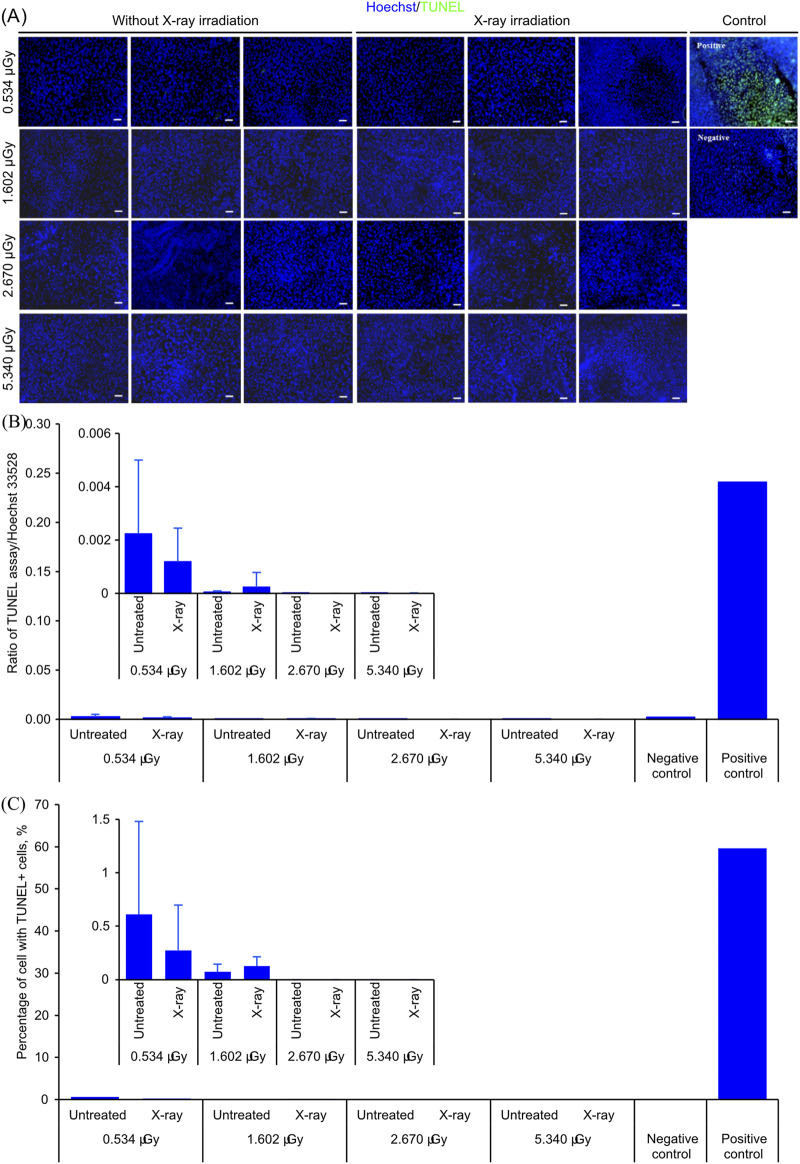
TUNEL assay of TEESs before and after various doses of X-ray irradiation. **(A)** Micrographs of TEESs before and after X-ray irradiation. The scale bars are 100 μm. **(B)** Ratio of fluorescence intensity of signal from TUNEL assay against Hoechst 33528 of TEESs before and after X-ray irradiation. Three biological replicates in each group. The plot represents mean ± standard deviation. **(C)** Percentage TUNEL+ cells from each sample before and after X-ray irradiation. Three biological replicates in each group. The plot represents the mean ± standard deviation. No statistical significance was detected based on Student’s t-test.

Figure 1 shows the results of the TUNEL assay after X-ray irradiation. No changes in the distribution of TUNEL^+^ cells were observed after X-ray irradiation (Figure 1A), suggesting that the dose of X-ray irradiation experienced by the samples might not induce DNA fragmentation. This observation was further confirmed by the ratio of fluorescence intensity of TUNEL against Hoechst 33258 measured by a spectrophotometer (Figure 1B). With a low significant difference of signal intensity, either analyzed under paired analysis or as batch samples, between the control and after X-ray irradiation at 0.534 μGy, 1.602 μGy, 2.670 μGy, and 5.34 μGy, it is unlikely that changes in DNA fragmentation were induced at 2 h after the dose of X-ray irradiation used in this experiment.

In prior studies conducted to understand the effect of low-dose X-ray irradiation on bone marrow mesenchymal stem cells, it was found that the highest number of DNA fragmentations in the nucleus was detected at 0.5 h after X-ray irradiation (Pustovalova et al., 2017). The number of DNA fragments decreased significantly at 4 h after irradiation. In this study, DNA fragmentation was measured 2 h after X-ray irradiation to optimize the detectability of DNA fragmentation and the characteristics of the TUNEL assay, which detects DNA fragmentation at its late stage, together with apoptosis.

To evaluate whether X-ray irradiation induces apoptosis, the ratio of TUNEL^+^ cells before and after X-ray irradiation was also measured (Figure 1C). Under all radiation doses tested in this study, the ratio of TUNEL^+^ cells was similar before and after X-ray irradiation, suggesting that the amount of X-ray irradiation used in this study might not induce additional cellular apoptosis.

### 3.2 Whole-exome sequencing of TEESs before and after X-ray irradiation

**FIGURE 2:**
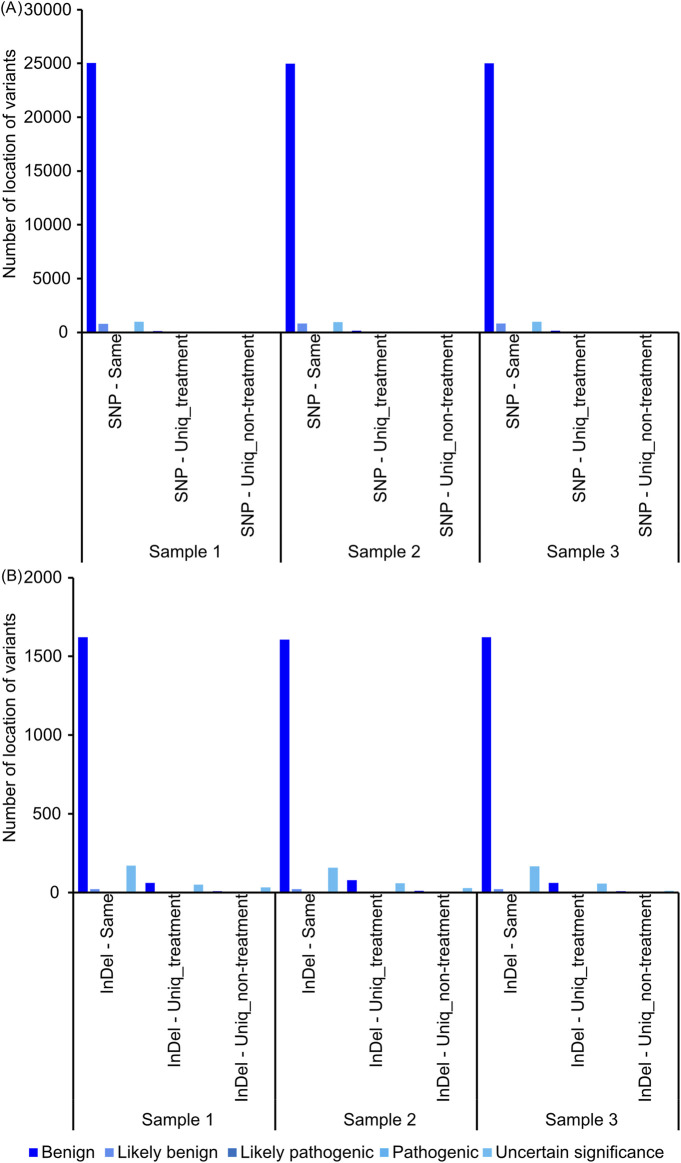
Number of variants obtained from exome sequencing after 5.340 μGy X-ray irradiation relative to control. **(A)** Number of locations of single-nucleotide polymorphisms of each sample after 5.340 μGy X-ray irradiation. **(B)** Number of locations of insertion and deletion of each sample after 5.340 μGy X-ray irradiation.

After incubation of TEESs with and without 5.340 μGy X-ray irradiation at 37 °C, humidified 5.0% CO_2_ incubator for 30 min, the samples were snap-frozen with liquid nitrogen before DNA extraction and whole-exome sequencing. As a single TEES was divided into several portions, and half of those portions were subjected to X-ray irradiation, it is possible to pair samples with and without X-ray irradiation by sample ID. Loci of variants were identified and mapped. Most of the variants detected were variants found commonly in both samples with and without X-ray irradiation (Figure 2), suggesting these mutations as background mutations specific to the donor. Single-nucleotide polymorphism (Figure 2A) or insertion/deletion (Figure 2B) variants detected in this study were benign, likely benign, or uncertain significant. Neither single-nucleotide polymorphisms nor insertions/deletions were detected as unique variants, shared among biological replicates (Supplementary Tables S1, S2), suggesting that 5.340 μGy X-ray irradiation might not contribute to X-ray irradiation-specific variants.

As the fixation of mutations generally occurs as a result of early damage, in this study, by detecting the immediate genomic response after X-ray irradiation, we attempt to cover both the early response and the mutations that follow. In addition, the detection coverage remained the same for all samples, which also supported that there was no change in the readable region in DNA, usually as a result of DNA fragmentation.

As shown in Figures 1A,B, 5.340 μGy X-ray irradiation did not induce DNA fragmentation. As X-ray-induced changes in DNA are usually the result of DNA breakage, without DNA fragmentation, it is possible that this dose of radiation does not induce specific mutations. Most of the variants found in exome sequencing are likely to be variants caused by background mutations, and these mutations are benign and distinct among samples.

### 3.3 X-ray irradiation does not affect the proliferative capabilities of cells

Another adverse effect of X-ray irradiation is alteration of proliferative capabilities of cells, either due to cell cycle arrest (Hirose et al., 2023) or uncontrolled proliferation (Sun et al., 2025). While DNA fragmentation was not detected, it is still important to confirm the proliferative capability of cells after X-ray irradiation to exclude the possibility of changes in proliferative capability due to undetected DNA fragmentation or mutation captured by exome sequencing.

**FIGURE 3:**
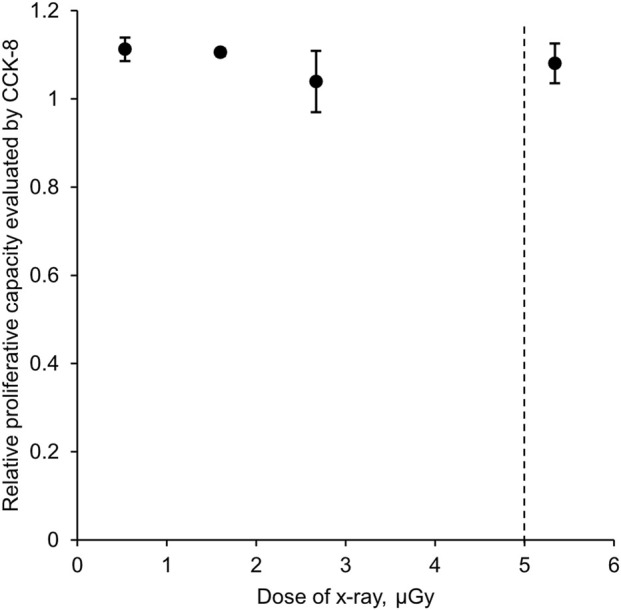
Proliferative capability of cells after X-ray exposure in relation to the control assessed by cellular activity. Three biological replicates in each group. The plot represents mean ± standard deviation. No statistical significance was detected based on Student’s t-test.

Figure 3 shows that the proliferative capability of cells after X-ray irradiation relative to the control group did not change within the range of X-ray irradiation tested. This result suggested that long-term cell cycle arrest or uncontrolled proliferation were not detected in our samples even after 5.340 μGy X-ray irradiation.

Unlike prior studies, which detected the effect of X-ray irradiation using bone marrow mesenchymal stem cells (Pustovalova et al., 2017) or hematopoietic stem cells (Petzer et al., 2002), TEESs contained various types of skin-derived cells, which included differentiated epidermal keratinocytes. Differentiated cells have low proliferative activity, as shown by their low expression of proliferation-related genes (Lian et al., 2024). Multiple passaging of cells introduces bias, as the resultant cells might be the products of basal cells with proliferative capability in TEESs. Therefore, it is more appropriate to evaluate the proliferative capability of cells digested from TEESs after X-ray irradiation.

As both cell cycle arrest and cellular senescence can be detected by a lower proliferative capability of cells, this result has also confirmed that neither cell cycle arrest nor cellular senescence was detected at this point of detection.

Nevertheless, it is important to note that the sample size of this study was small. A more statistically sound conclusion can likely be produced by using a larger sample size, that is, more TEESs cultured from cells obtained from more donors. However, consider that the sample was precious as the cells have limited proliferative capacity, and the needs of patients should be prioritized over this study. Therefore, in this study, TEESs produced from three donors were used to rule out biological variation against X-ray irradiation.

In addition, in our study, a piece of TEES was split into two pieces, subjected to 5.340 μGy X-ray irradiation, and transported with a cold-chain courier service at 4 °C for 1.5 days. The proportion of melanocytes after X-ray irradiation and transportation was 4.00% against 3.85% before transportation. As the proportion of melanocytes and the integrity of TEESs are important factors in achieving biological functionality of TEESs, and altering the function of TEES will result in a loss of melanocytes, it is likely that X-ray irradiation did not produce a lethal effect on TEESs.

In this study, we have shown that repeated exposure of TEESs to X-rays emitted by a luggage-control system, even to a maximum dose limited by regional regulations, did not induce changes in TEESs. This might simplify the transportation and reduce the cost of grafts.

## Data Availability

The original contributions presented in the study are included in the article/Supplementary Material; further inquiries can be directed to the corresponding author.
